# Laparoscopic Management of Small Bowel Obstruction in a Patient With Prior Kidney and Pancreas Transplantation

**DOI:** 10.7759/cureus.106156

**Published:** 2026-03-30

**Authors:** David Roberts, Olivia Haney, Lisa Y Shimotake, Indraneil Mukherjee

**Affiliations:** 1 Surgery, Stony Brook University, Stony Brook, USA; 2 General Surgery, Northwell Health Staten Island University Hospital, Staten Island, USA

**Keywords:** closed loop obstruction, laparoscopic small bowel resection, simultaneous pancreas and kidney transplantation, small-bowel obstruction, surgical or open or laparoscopic and adhesive small bowel obstruction or intestinal obstruction

## Abstract

Simultaneous pancreas-kidney (SPK) transplantation is a surgical treatment for patients with long-standing type 1 diabetes mellitus who consequently develop diabetic nephropathy and end-stage renal disease. Well-documented complications of pancreas transplant surgery include acute rejection, chronic rejection, pancreatitis, graft thrombosis, hemorrhage, anastomotic leakage, and intra-abdominal infection. However, small bowel obstruction (SBO), either through internal hernias or adhesive bands, can also occur. Our report details the case of a patient with a history of SPK transplantation 1.5 years earlier, presenting with peritonitis on examination and CT imaging concerning for a closed-loop SBO between the pancreatic allograft and small bowel mesentery. The patient successfully underwent laparoscopic management with laparoscopic small bowel resection and primary anastomosis.

## Introduction

Simultaneous pancreas-kidney (SPK) transplantation is a surgical treatment for patients with long-standing type 1 diabetes mellitus who consequently develop diabetic nephropathy [1]. SPK accounts for 83% of pancreas transplant procedures, while only 12% are two-stage procedures in which pancreas transplantation follows kidney transplantation. SPK is known to reduce morbidity in these patients and improve cardiovascular mortality compared to live donor kidney transplantation [2, 3]. The mean survival for patients who underwent SPK was 23.4 years, compared to 12.9 years in those who underwent pancreas transplantation alone [1]. The US Department of Health and Human Services Organ Procurement and Transplantation Network reports that more than 26,000 SPK transplantations have been performed in the United States since 1988 [4]. These patients tend to have successful outcomes. A single-center study in the UK followed 193 patients who underwent SPK between 1996 and 2010. The study showed that at 1- and 5-year follow-up, pancreas graft survival was 89% and 80%, kidney graft survival was 93% and 83%, and patient survival was 97% and 92%, respectively [5]. In that same study, 44 of the 193 patients experienced surgical complications that required re-laparotomy within the first 3 months, and only 1 of those complications was due to small bowel obstruction (SBO). Few studies have looked specifically at SBO in the setting of prior SPK. One such report suggests that, when followed over a moderate time period (~2 years), as many as 10% of patients may develop SBO, most commonly due to adhesions or internal hernias [6]. No studies have evaluated the management of such obstructions. Our report details the laparoscopic management of a closed-loop SBO that required bowel resection in a patient who had undergone SPK transplantation 1.5 years earlier.

## Case presentation

**Figure 1:**
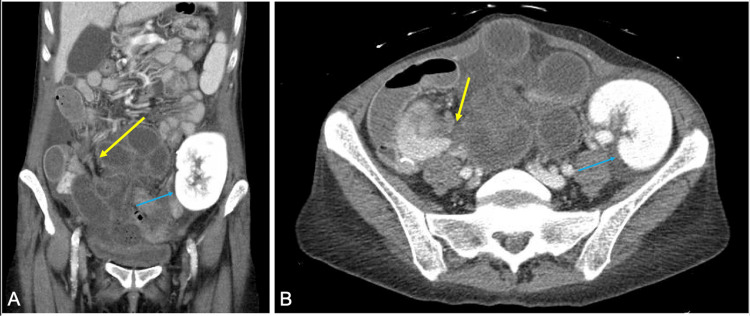
(A) Coronal and (B) axial views of CT of the abdomen and pelvis showing the transition point of a closed-loop small bowel obstruction. Yellow arrow: adhesive band; Light blue arrow: renal allograft.

The patient was a 47-year-old female with a history of type 1 diabetes mellitus (diagnosed at age 20, on insulin, hemoglobin A1c 7.2), uncontrolled hypertension, HIV (on Descovy and Tivicay), and end-stage renal disease on hemodialysis through a left upper extremity arteriovenous fistula. Her last colonoscopy and esophagogastroduodenoscopy were reportedly normal. The patient underwent simultaneous left kidney and pancreas transplantation, which was complicated postoperatively by poorly controlled hypertension requiring a nicardipine drip, ileus requiring nasogastric tube placement, and a hemoglobin drop to 6.0 requiring 2 units of red blood cells, all of which resolved prior to discharge. The patient presented 1.5 years later with a 1-day history of severe epigastric abdominal pain radiating to the bilateral lower quadrants, along with nausea and 8 episodes of bilious emesis. At that point, she had no longer tolerated any oral intake for about 24 hours. On physical examination, the patient was in acute distress with bilateral lower quadrant tenderness, guarding, and rebound tenderness, findings consistent with peritonitis. CT of the abdomen and pelvis showed tethering of multiple loops to a single point in the right lower quadrant. Additionally, abdominopelvic ascites and mesenteric edema were appreciated (Figure 1). Laboratory findings were grossly normal on presentation, outside of hemoconcentration seen on the complete blood count, likely due to dehydration (Table 1). Of note, the normal lactate was likely due to this being a closed-loop obstruction. In closed-loop obstructions, twisting of the mesentery can prevent venous outflow from the ischemic bowel. Given the urgent nature of surgery, CD4 count and HIV status were not evaluated preoperatively.

**Table 1:** Pertinent laboratory findings on presentation to the ED.

Lab finding	Result	Normal value range
WBC count (K/µL)	11.74 (H)	4.80-10.80
Hemoglobin (g/dL)	18.4 (H)	14.0-18.0
Glucose (mg/dL)	104 (H)	70-99
Blood urea nitrogen (BUN) (mg/dL)	13	5-20
Creatinine (mg/dL)	0.8	0.5-1.2
Lactate (mmol/L)	1.3	0.5-2.2

**Figure 2:**
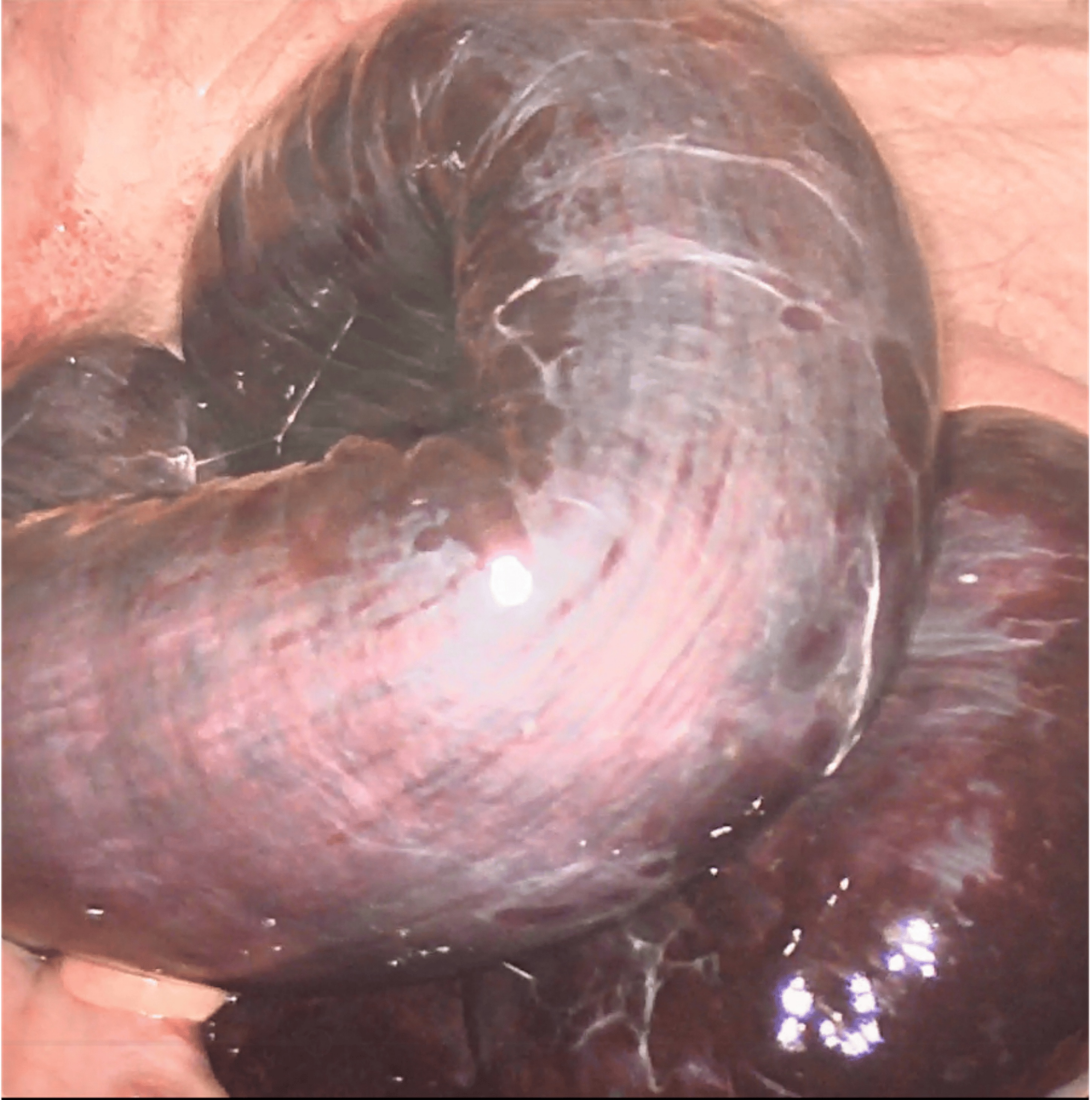
Laparoscopic view of ischemic small bowel.

**Figure 3:**
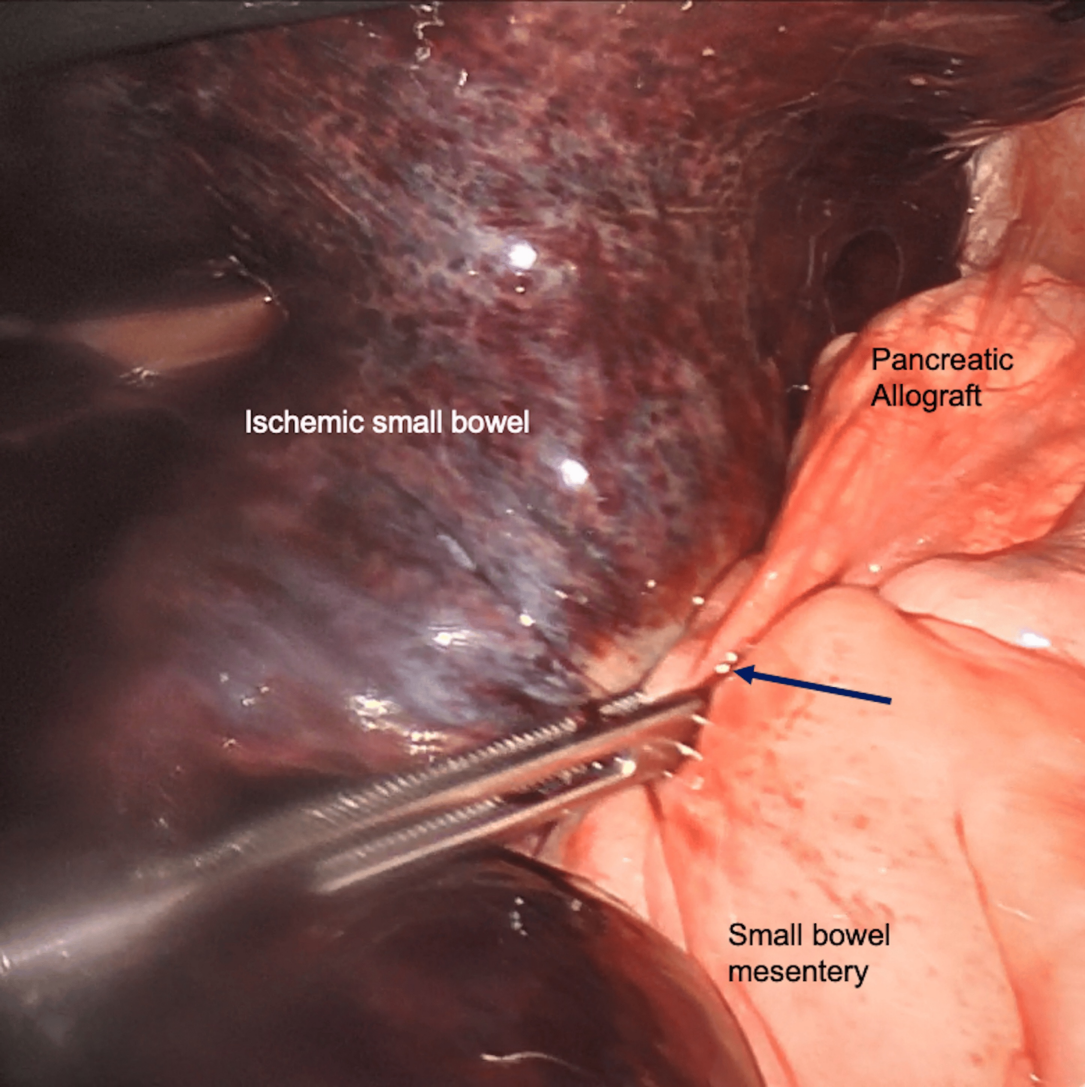
Laparoscopic view of the adhesive band extending from the pancreatic allograft to the small bowel mesentery. Dark blue arrow: adhesive band.

The patient underwent urgent diagnostic laparoscopy, which revealed grossly necrotic small bowel from the distal ileum to the cecum (Figure 2). One adhesive band extending from the pancreatic allograft to the small bowel mesentery was noted to be causing a closed-loop SBO (Figure 3). The pancreatic allograft with duodenal cuff, as well as the anastomosis to the jejunum, was viable and intact. A laparoscopic resection of the distal ileum and cecum measuring 45 centimeters (cm) in length was performed with a stapled side-to-side ileocolic anastomosis using a 60-millimeter (mm) long 4.0 mm staple load. The common channel of the anastomosis was then closed laparoscopically using a 9-inch 2-0 absorbable braided suture. Case duration was 221 minutes. Estimated blood loss was minimal. The patient was admitted to the surgical step-down unit and maintained a nasogastric tube postoperatively. She regained bowel function on postoperative day 3, the nasogastric tube was removed, and she was eventually progressed to a low-residue diet. She was continued on her home medications, including Descovy and Tivicay, in the acute postoperative period. Once her pain improved, she was safely discharged from the hospital 1 week following her initial presentation. She has had no other episodes of bowel obstruction since this initial presentation.

## Discussion

SBOs are relatively common complications of abdominal surgery, with an overall incidence of 4.6% [6]. However, this incidence rate is highly dependent on the type of procedure performed, with open procedures having higher rates than those performed laparoscopically [6]. Management of SBO thus depends on the etiology and severity.

SBO with features of bowel compromise is an emergency and requires immediate surgical intervention. Management of SBO depends on the etiology and severity. If the bowel is not compromised, then reduction back to the anatomical position with closure of the fascial defect or simply lysis of adhesions may be appropriate. For patients whose bowel appears to be ischemic beyond the point of viability, management includes resection of the ischemic portion of small bowel with either primary anastomosis or diversion in some extreme cases. The conventional immediate surgical method for SBO is an open exploratory laparotomy; however, advancements in minimally invasive techniques have allowed laparoscopy to become an increasingly beneficial option for treatment in these acute care patients.

Studies evaluating laparoscopic compared to open management of bowel obstruction have shown mixed evidence. Recent studies have shown that patients who successfully undergo laparoscopic management of SBO have significantly shorter lengths of hospital stay and decreased overall morbidity; however, conversion to open surgery can occur in over 40% of cases [7]. Further studies have shown a shorter return of bowel function with adhesiolysis alone; however, when bowel resection and anastomosis occur, there is no difference between laparoscopic and open approaches. However, the ultimate factor determining the success of laparoscopic management is patient selection and surgeon preference [8].

The documented causes of SBO in patients with SPK transplantation are adhesions and internal hernias [9]. A retrospective analysis that looked at 99 enteric drainage pancreas transplantations over a 2-year period found that 19 patients (19.4%) developed bowel complications. Ten of the 19 patients (10.2%) developed high-grade SBO: 3 due to internal hernias and 7 due to adhesions [9]. Internal hernias can occur through the defect that is purposely made in the mesentery during surgery to allow the donor duodenum/pancreas allograft to access the recipient’s jejunum. The defect is susceptible to bowel entering the space posterior to the donor allograft and anterior to the posterior wall of the peritoneal cavity, thus causing an obstruction [9]. Moreover, SBO can form due to adhesions that develop as part of a local inflammatory response to surgery. In SPK transplant procedures, the pancreas is traditionally placed within the intraperitoneal cavity in the right iliac fossa, and the kidney is placed in the extraperitoneal space in the left iliac fossa. Ipsilateral implantation has also been found to be safe. Given that the pancreatic allograft is within the peritoneal cavity, it is possible for surgical adhesions to form, which can lead to SBO in these patients [10].

Our patient had laboratory findings of mild leukocytosis and mildly elevated lactate levels. However, the patient’s physical examination showed signs of peritonitis, and CT imaging demonstrating mesenteric edema and ascitic fluid was consistent with bowel compromise and concern for a closed-loop obstruction, warranting intervention. The obstruction that occurred in our case was due to an adhesive band extending from the pancreatic allograft to the small bowel mesentery. CT imaging showed a fibrous band extending from the pancreatic allograft and distended small bowel segments located in the anterior aspect of the abdominal cavity (Figure 1). Upon entry into the abdomen, frankly necrotic bowel (Figure 2) was appreciated, with segments of collapsed small bowel both proximal and distal to the area of concern. The adhesive band was identified (Figure 3) and resected laparoscopically, allowing us to appreciate healthy portions of small bowel proximally and distally and to safely complete the laparoscopic resection and primary anastomosis.

Of note, pancreatic transplant studies may not report SBO as a common surgical complication because of lag time in reporting. While other studies often evaluate surgical complications within the first 90 days, the clinical presentation of an SBO often occurs much later, ranging from months to years or even decades. In a few studies that followed patients to 1 year postoperatively, the mean presentation of an SBO due to adhesions was 170 days after surgery, with 21 days being the earliest presentation reported. Additionally, obstructions secondary to internal hernias were reported on average between 57 and 367 days postoperatively in those same studies [9]. Other documented complications of pancreas transplant surgery include acute rejection, chronic rejection, pancreatitis, graft thrombosis, hemorrhage, anastomotic leakage, and intra-abdominal infection [1,11].

## Conclusions

Our case highlights a patient with a history of type 1 diabetes mellitus, hypertension, HIV, end-stage renal disease, and prior SPK transplantation who presented with a closed-loop SBO. A literature search revealed that SBO is a rare finding among postoperative patients who experience surgical complications following pancreas transplantation. Minimally invasive management of this complication is difficult. We hope to provide an example of a patient who experienced a closed-loop bowel obstruction after previously undergoing SPK transplantation and who was managed laparoscopically with small bowel resection and primary anastomosis. Therefore, laparoscopic management of adhesive SBO may be considered in this patient population in carefully selected patients by experienced minimally invasive surgeons.
